# Multi-Omics Insights into the Relationship Between Intestinal Microbiota and Abdominal Fat Deposition in Meat Ducks

**DOI:** 10.3390/ani15233393

**Published:** 2025-11-24

**Authors:** Zhixiu Wang, Chunyan Yang, Yan Li, Bingqiang Dong, Qianqian Song, Hao Bai, Yong Jiang, Guobin Chang, Guohong Chen

**Affiliations:** 1 Key Laboratory for Animal Genetics & Molecular Breeding of Jiangsu Province, College of Animal Science and Technology, Yangzhou University, Yangzhou 225009, China; wangzx@yzu.edu.cn (Z.W.); yangcy202109@163.com (C.Y.); bqdong2021@163.com (B.D.); songqianqian8sdau@163.com (Q.S.); bhowen1027@yzu.edu.cn (H.B.); jiangyong@yzu.edu.cn (Y.J.); gbchang@yzu.edu.cn (G.C.); 2 Livestock Development and Promotion Center of Pingyi County, Linyi 276799, China; pyliyan001@163.com

**Keywords:** 16S rRNA gene, metagenome, abdominal fat deposition, meat duck, intestinal microbiota

## Abstract

Abdominal fat deposition is an important economic trait in poultry, as excessive accumulation reduces feed efficiency and carcass yield. The gut microbiota is known to influence host energy metabolism and fat storage, suggesting its potential involvement in fat deposition. This study examined the relationship between intestinal microbiota and abdominal fat deposition in an F_2_ population derived from Cherry Valley Ducks (♂) × Runzhou Crested White Ducks (♀) at 42 days of age. Based on abdominal fat rate, ducks with values of 0–0.75% and 1.5–2.25% were defined as the low (LF) and high (HF) abdominal fat groups, respectively. A combined multi-omics approach was used, including 16S rRNA gene sequencing, metagenomics, and whole transcriptomics, to compare high and low abdominal fat rate groups. 16S rRNA gene sequencing results showed that the cecum had the highest microbial diversity among all intestinal segments (duodenum, jejunum, ileum, and rectum) and was significantly enriched in carbohydrate metabolism pathways, highlighting its key role in nutrient utilization and growth. Therefore, the cecum was selected for further analysis. Metagenomic analysis of the cecum contents revealed significantly different intestinal microbial β diversity between the high and low abdominal fat rate groups (*p* < 0.05). The low abdominal fat rate group was enriched in beneficial microorganisms such as *Paenibacillus*, *Butyrivibrio*, *Coprococcus*, Ruminococcaceae, Veillonellaceae (Clostridiales), and Firmicutes. Conversely, the high abdominal fat rate group was characterized by an increased abundance of Bacteroidetes, including both beneficial and potentially pathogenic taxa such as *Alistipes* and Eggerthellales. The integrated analysis of metagenomic and whole transcriptome sequencing showed that Firmicutes and Bacteroidetes were not only related to energy metabolism, lipid metabolism, and amino acid metabolism, but also to the expression of *FGF2*, *FKBP5*, *PNPLA2*, *PLIN3*, *FGFR2*, *DGAT2*, and *ACER2*. In addition, Firmicutes and Bacteroidetes were also associated with 7 lncRNAs: XR_003493494.1, XR_003492471.1, XR_001190174.3, TCONS_00005095, XR_001190238.3, TCONS_00005095, and XR_003492841.1. In conclusion, this study highlights that the cecal microbiota is closely associated with abdominal fat deposition in ducks, elucidating its potential influence on host metabolism and gene expression. These findings enhance our understanding of the gut microbiota’s relationship with obesity and offer new strategies to modulate gut–microbe interactions to reduce abdominal fat accumulation in poultry.

## Simple Summary

Excessive abdominal fat deposition in meat ducks not only reduces feed efficiency but also affects carcass quality, posing a challenge to the meat duck industry. Increasing evidence suggests that intestinal microbiota can influence fat metabolism and energy storage in animals. In this study, we explored the relationship between ducks with high and low abdominal fat rate and their intestinal microbiota in different segments. Using Multi-Omics analyses, we found that the cecum plays a crucial role in nutrient utilization and growth in ducks. Ducks with low abdominal fat rate had more beneficial bacteria that help digest carbohydrates and produce short-chain fatty acids, which can reduce fat deposition. In contrast, ducks with high abdominal fat rates had more bacteria linked to inflammation and poor metabolism. Further analysis revealed that the intestinal microbiota is associated with the expression levels of genes and lncRNAs related to abdominal fat production and energy utilization. These findings highlight a strong association between intestinal microbiota and fat deposition, providing a scientific basis for strategies such as targeted feeding or microbiome modulation to optimize fat distribution, enhance feed efficiency, and improve meat quality in meat ducks.

## 1. Introduction

Duck meat is a popular source of protein. Long-term intensive genetic selection and large-scale breeding have improved the feed conversion efficiency, growth rate, and meat yield of modern meat ducks; however, they also increase abdominal fat content and reduce intramuscular fat deposition [1]. Excessive abdominal fat deposition adversely affects feed conversion efficiency and carcass yield [2,3]. An appropriate increase in intramuscular fat content during duck production can improve meat quality, including tenderness, flavor, and juiciness [4,5]. Therefore, reducing abdominal fat deposition and increasing intramuscular fat deposition are major concerns in meat duck breeding worldwide. Fat deposition is a complex process regulated by nutrition and multiple transcription factors, fat-related genes, and signaling pathways [6]. Moreover, the intestinal microbiota is an important factor influencing fat deposition [7,8]. Alterations in certain intestinal microorganisms can lead to chronic metabolic disorders, including obesity [9,10,11].

The animal intestinal microbiota is a complex and dynamic ecosystem inhabited by trillions of diverse symbiotic microbial communities. Within the intestine, the microbiota contributes to immune system development, metabolism, and barrier defense against pathogens, but it also harbors opportunistic or pathogenic bacteria that can impair intestinal health when microbial balance is disturbed [12,13]. Meanwhile, changes in intestinal function directly affect lipid metabolism, potentially leading to obesity. Microorganisms inhibit the intestinal adipocytokines to promote obesity [14]. Moreover, fecal transplantation of intestinal microbiota from obese humans, mice, or pigs to sterile or antibiotic-treated mice promotes fat deposition [15,16,17]. Wen et al. [18] identified two microbial taxa, *Methanobrevibacter* and *Mucispirillum schaedleri*, that are substantially associated with fat deposition in the cecum of broiler chickens. Meanwhile, Lyu et al. [19] reported that *Treponema* and *Ruminococcus torques* are markedly associated with fat deposition in ducks.

Cherry Valley Ducks are fast-growing, large-sized commercial meat ducks characterized by rapid growth, high meat yield, and significant subcutaneous and abdominal fat deposition. They are widely used in meat production, especially in roast duck processing [20]. In contrast, Runzhou Crested Ducks grow more slowly and have lower abdominal fat deposition. This breed is valued for both its ornamental and economic worth, and is highly sought after due to its traditional medicinal value [21]. Crossbreeding Cherry Valley Ducks with Runzhou Crested Ducks combines the former’s rapid growth with the latter’s high nutritional value, resulting in F_2_ generations with significantly different abdominal fat deposition. This population is an ideal model for studying the relationship between duck gut microbiota and abdominal fat.

However, most studies have focused on chickens, and the mechanisms linking intestinal microbiota and lipid deposition in ducks remain largely unexplored. Ducks differ from chickens in intestinal physiology and microbial composition, including longer intestinal transit time and higher intestinal water content, which may shape distinct microbial community structures [22]. The duck gut microbiota is dominated by Firmicutes, Proteobacteria, Actinobacteria, and Bacteroidetes [23], whereas that of chickens is mainly dominated by Firmicutes and Bacteroidetes with a higher Firmicutes/Bacteroidetes ratio [24]. These differences highlight the need to investigate duck-specific microbial mechanisms related to fat deposition.

The current study explores the relationship between intestinal microbes and abdominal fat deposition in ducks by analyzing the host phenotype, whole transcriptome, intestinal 16S rRNA gene sequencing, and cecum content macrogenome of a 42-day-old F_2_ generation Cherry Valley Duck (♂) × Runzhou Crested White Duck (♀). The findings provide insights for developing new strategies targeting intestinal-microbe interactions to reduce abdominal fat accumulation in meat ducks.

## 2. Materials and Methods

### 2.1. Experimental Animals

The F_2_ generation of 304 Cherry Valley Duck (♂) × Runzhou Crested White Duck (♀) was purchased from Shuyang Zhongke Seed Poultry Co., Ltd. (Suqian, Jiangsu, China). All ducks were reared in a uniform environment up to 42 days of age, during which they had free access to food and water. All ducks were fed the same commercial basal diet formulated according to the Feeding Standard of Meat-type Ducks (NY/T 2122-2012, Agricultural Industry Standards of the People’s Republic of China). The compositions and nutrients of the experimental diets are presented in Table 1. Ducks were housed in well-ventilated rooms maintained at a temperature of 28–33 °C with 60–70% relative humidity and a 16 h light/8 h dark photoperiod. All experimental ducks were managed and handled following the guidelines approved by the Animal Care and Use Committee of Yangzhou University (No. YZUDWSY2017-11-07). Ducks were slaughtered at 42 days of age. After fasting for 8 h, the ducks were slaughtered in a stun bath (900 Hz, 40 V) for 5 s, and the jugular vein and carotid artery on one side of the neck were severed and exsanguinated. They were then slaughtered and plucked. The weight of the defeathered carcass was determined as the carcass weight (CW). The carcass was then manually eviscerated, weighed after removing viscera, including the crop, trachea, esophagus, spleen, pancreas, gallbladder, gonads, and intestinal tract, which was recorded as semieviscerated weight (SEW). The eviscerated weight (EW) was measured as SEW after removing the heart, liver, gizzard, proventriculus, and abdominal fat. The weight of abdominal fat is abbreviated as AFW. The molecular samples were washed in PBS (Procell, Wuhan, China), and then excess water was removed. The abdominal fat rates were determined using AFW/(AFW + EW) × 100%. The 42-d F_2_ generation ducks with abdominal fat rates of 0–0.75% and 1.5–2.25% were assigned to the low abdominal fat rate (LF) and high abdominal fat rate (HF) groups, respectively. The duodenum (SC-L), jejunum (KC-L), ileum (HC-L), cecum (MC-L), and rectum (ZC-L) of six male ducks randomly selected from the LF group and HF group (SC-H, KC-H, HC-H, MC-H, and ZC-H, respectively) were subjected to 16S rRNA gene sequencing. Based on 16S rRNA results, three representative cecal samples from each group (L-MC for LF and H-MC for HF) were further selected for metagenomic sequencing. The selection was made according to microbial community composition, diversity indices, and clustering patterns derived from the 16S rRNA data. This design aimed to balance sequencing depth, cost, and biological representativeness.

### 2.2. Extraction of Genomic DNA

Genomic DNA was extracted from different intestinal segments of ducks using the Stool Genomic DNA Extraction Kit (Solarbio, Beijing, China) according to the manufacturer’s instructions. NanoDrop microspectrophotometry (NanoDrop 2000, Thermo Fisher Scientific, Waltham, MA, USA) was used to detect the optical density of nucleic acids; the A260/A280 ratio was between 1.8 and 2.0, and the A260/A230 ratio was approximately 2.2. Agarose gel electrophoresis was performed to assess the quality of the genomic DNA.

### 2.3. 16S rRNA Gene Sequencing

The V3-V4 region of the bacterial 16S rRNA gene was amplified using primers 341F (CCTACGGGNGGCWGCAG) and 806R (GGACTACHVGGGTATCTAAT) containing unique barcodes. PCR amplification products were recovered by gel electrophoresis and quantified using a QuantiFluor TM fluorometer (Promega, Madison, WI, USA). Purified amplification products were mixed in equal amounts, sequencing adapters were ligated, sequencing libraries were constructed according to the instructions provided by Illumina, and sequencing was performed on a Hiseq2500 platform in the PE250 mode.

### 2.4. 16S rRNA Gene Data Processing and Analysis

Raw data were quality-controlled using FASTQ to obtain clean reads, which were spliced at both ends using FLASH software (v. 1.2.11) [19]. The tag sequences were de-redundantly processed using the Mothur (v. 1.39.1) software package, from which unique tag sequences were selected. Operational taxonomic units (OTUs) with ≥97% similarity were clustered using UPARSE (usearch v. 9.2.64) [25]. Representative sequences were biologically classified based on the SILVA database (https://www.arb-silva.de/, accessed on 22 August 2023) using the RDP Classifier (v. 2.2) and a Naive Bayesian model; the classification confidence threshold ranged from 0.8 to 1. Species identification and annotation were performed at the kingdom, phylum, class, order, family, genus, and species levels using Krona (v. 2.6) [26]. Species comparison between groups was calculated by Welch’s *t*-test and Wilcoxon rank test in the R project Vegan package (version 2.5.3) [27]. Biomarker profiles for each group were screened using Metastats (v. 20090414) [28] and linear discriminant analysis (LDA) effect size (LEfSe) (v. 1.0) [29] software packages. The LDA score threshold was set to >2.5 and *p* < 0.05 as the significance criterion. The Kyoto Encyclopedia of Genes and Genomes (KEGG) pathway analysis of OTUs was inferred using Tax4Fun (version 1.0) [30]. Analysis of functional differences between groups was calculated by Welch’s *t*-test in the R project Vegan package (version 2.5.3) [27].

### 2.5. Metagenomic Library Construction and Sequencing

DNA from cecal contents was extracted using HiPure Bacterial DNA Kits (Magen, Guangzhou, China). DNA quality was determined using agarose gel electrophoresis and NanoDrop microspectrophotometry (NanoDrop 2000). The NanoDrop assay required a sample volume of 2 μL and measured concentrations of 2–3000 ng/μL. The A260/A280 ratio for nucleic acids was 1.8–2.0. Agarose gel electrophoresis showed that the nucleic acid samples were not degraded and had no protein or other contamination. Qualified DNA samples were randomly broken into 350 bp fragments using an ultrasonic disruptor (Covaris, Woburn, MA, USA), and the entire library was prepared by end repair. A-tail addition, sequencing adapter addition, purification, and PCR amplification were performed. Following library construction, quantification, and testing were performed using a QuantiFluorTM fluorometer (Promega), and the qualified libraries were subjected to Illumina onboard sequencing.

### 2.6. Metagenomic Sequencing Data Processing and Analysis

Fastp (v. 0.18.0) was used to obtain clean data for subsequent analysis; the filtered data were aligned to the host reference genome using Bowtie2 (v. 2.2.5) [31,32]. Reads derived from the host were filtered to obtain effective reads, which were then assembled using the MEGAHIT (v. 1.1.2) software [33]. Genes >500 bp were predicted using MetaGeneMark (v 3.38). CD-HIT (v. 4.6) software (95% identity, 90% coverage) was used to cluster the predicted genes [34,35], and the longest genes were selected as representative sequences of each class to construct an initial non-redundant gene collection. Clean reads were realigned to the initial non-redundant gene set using Bowtie2. Based on the alignment results, reads were reassigned to the best gene using the Pathoscope (v. 2.0.7) software. Genes with ≤2 reads supported in each sample were filtered to obtain the final gene set for subsequent analysis. The unigenes were annotated using DIAMOND [34] (version 0.9.24) by aligning with the deposited data in KEGG and evolutionary genealogy of genes: Non-supervised Orthologous Groups (eggNOG). Additional annotation was based on the Carbohydrate-Active enZYmes (CAZy) database. The predicted genes were mapped to NCBI non-redundant genome databases using DIAMOND [36] (version 0.9.24). The alignment results were submitted to MEGAN (version 6.19.9) [37] to estimate the taxonomic compositions with the weighted lowest common ancestor (LCA) algorithm [38]. Species/function comparisons between and among groups were calculated by Welch’s *t*-test and ANOVA (analysis of variance), respectively, in the R project Vegan package.

### 2.7. Diversity Analysis

Chao1, abundance-based coverage estimators (ACEs), Shannon, and Simpson indices were calculated using the Python scikit-bio package (version 0.5.6). The Alpha index comparison between groups was calculated by Welch’s *t*-test in the R project Vegan package. The Bray–Curtis distance matrix based on gene/taxon/function abundance was generated by the R Vegan package. Multivariate statistical techniques, including principal component analysis (PCA) and principal coordinates analysis (PCoA) of Bray–Curtis distances, were calculated using the R vegan package and plotted using the R ggplot2 package.

### 2.8. Combined Metagenomic and Whole Transcriptome Analysis

The metagenome and whole transcriptome association analysis was mainly performed through differential genes, differential lncRNAs, intestinal flora, and pathways. First, the Pearson correlation coefficient was calculated for the intestinal bacteria screened by metagenomic sequencing and the differential genes screened by the whole transcriptome using the R psych package (version 1.8.4). Secondly, the Pearson correlation coefficient was calculated for the pathways screened by metagenomic sequencing and the differential genes screened by the whole transcriptome using the R psych package (version 1.8.4). Spearman correlation analysis was performed between the abundance of intestinal microbial taxa and the expression levels of host genes and lncRNAs. Correlations with an absolute correlation coefficient (|r|) greater than 0.9 and a *p* < 0.05 were retained. The selected genes, lncRNAs, intestinal flora, and metabolic pathways were then visualized using Cytoscape software (version 3.9.1).

### 2.9. Statistical Analysis

All abdominal fat rate data were imported into Microsoft Excel 2019, and results are expressed as mean ± standard error of the mean (SEM). Statistical significance between groups was evaluated using a two-sample *t*-test conducted in SPSS 16.0 (SPSS Inc., Released 2007, SPSS for Windows, Version 16.0, Chicago, IL, USA). A *p* ≤ 0.05 was considered statistically significant.

## 3. Results

### 3.1. Statistical and Diversity Analysis of OTUs

**Figure 1:**
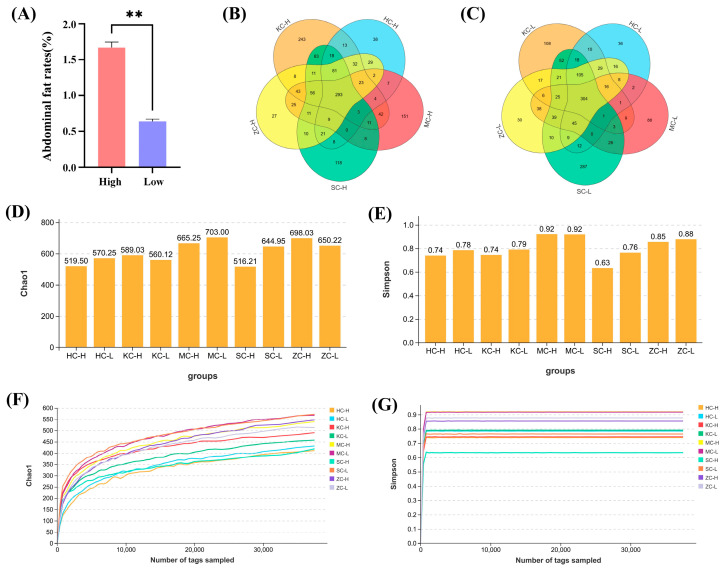
Statistical and diversity analyses of OTUs: (**A**) Statistics of high and low abdominal fat rate. ** represents *p* < 0.01. (**B**) Venn diagram of unique and shared OTU statistics in the five intestinal segments of the high abdominal fat rate group. (**C**) Venn diagram of unique and shared OTU statistics in the five intestinal segments of the low abdominal fat rate group. (**D**,**E**) Histogram of Chao1 and Simpson diversity indexes for five intestinal segments of each sample. (**F**,**G**) Chao1 and Simpson diversity index rarefaction curves for five intestinal segments per sample.

A previous comparison of abdominal fat rate in 304 ducks revealed considerable differences among individuals [6]. To investigate the relationship between high and low abdominal fat rates and intestinal microbiota, six ducks were selected from the HF and LF groups for 16S rRNA gene sequencing. The raw 16S rRNA gene sequence data quality control results revealed an efficiency rate of >90% (Supplementary Table S1). The average abdominal fat rates for ducks in the HF and LF groups were 1.67% and 0.64%, respectively (Figure 1A). Significant differences were detected in the OTUs within the five gut segments (Supplementary Tables S2 and S3). Venn diagrams were created to distinguish between shared and unique OTUs in the test groups. All intestinal segments exhibited a high degree of microbial species richness and diversity (Figure 1B,C). Meanwhile, no significant differences were observed in species diversity based on the Chao1 and Simpson indices between the two groups among all five intestinal segments (Figure 1D–G; *p* > 0.05).

### 3.2. Dominant Intestinal Flora

**Figure 2:**
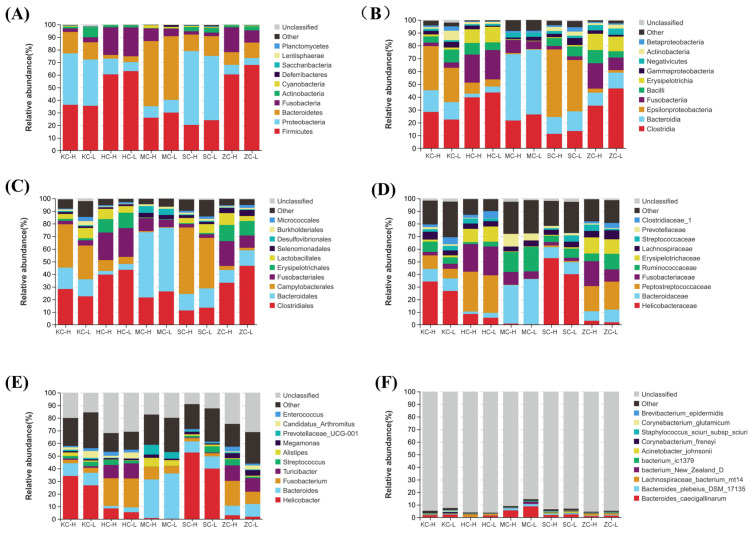
Gut microbiota composition of the duodenum, jejunum, ileum, cecum, and rectum in the high and low abdominal fat rate groups: (**A**) Composition of the intestinal microbiota of the duodenum, jejunum, ileum, cecum, and rectum at the phylum level. (**B**) Composition of the intestinal microbiota of the duodenum, jejunum, ileum, cecum, and rectum at the class level. (**C**) Composition of the intestinal microbiota of the duodenum, jejunum, ileum, cecum, and rectum at the order level. (**D**) Composition of the intestinal microbiota of the duodenum, jejunum, ileum, cecum, and rectum at the family level. (**E**) Composition of the intestinal microbiota of the duodenum, jejunum, ileum, cecum, and rectum at the genus level. (**F**) Composition of the intestinal microbiota of the duodenum, jejunum, ileum, cecum, and rectum at the species level.

The Pearson correlation analysis results (Supplementary Figure S1) and species distribution (Supplementary Figure S2) among different bacterial taxa showed that the gut microbiota with each other co-regulate the development of the organism. To determine the differences in intestinal flora between the HF and LF groups, the abundance plots were analyzed at the kingdom, phylum, class, order, family, genus, and species levels. At the phylum level, Firmicutes were more abundant in all intestinal segments, excluding the jejunum, of the LF group compared with the HF group (Figure 2A). At the class level, Bacilli, Actinobacteria, and Gammaproteobacteria were less abundant in the jejunum of the HG group compared with the LF group. Meanwhile, jejunal Epsilonproteobacteria, Bacteroidia, and Clostridia were slightly more abundant in the LF group than in the HF group (Figure 2B). At the order level, the abundance of Lactobacillales in the jejunum and duodenum was lower in the HF group than in the LF group. In contrast, the opposite pattern was observed in the ileum and rectum (Figure 2C). At the family level, Clostridiaceae_1 was more abundant in the LF group than in the HF group in the jejunum, ileum, and rectum. In the cecum, Bacteroidaceae were the most abundant in both HF and LF, while Helicobacteraceae were the least abundant (Figure 2D). At the genus level, *Bacteroides* in both HF and LF were widely distributed in the cecum, while *Helicobacter* predominated the jejunum and duodenum. Moreover, the abundance of *Alistipes* and *Fusobacterium* was higher in the cecum of the HF group than in the LF group (Figure 2E). At the species level, *bacterium_New_Zealand_D*, *bacterium_ic1379*, and *Bacteroides caecigalinarum* were most enriched in the cecum, and were more abundant in the LF group than in the HF group (Figure 2F).

### 3.3. Significantly Differential Microflora

**Figure 3:**
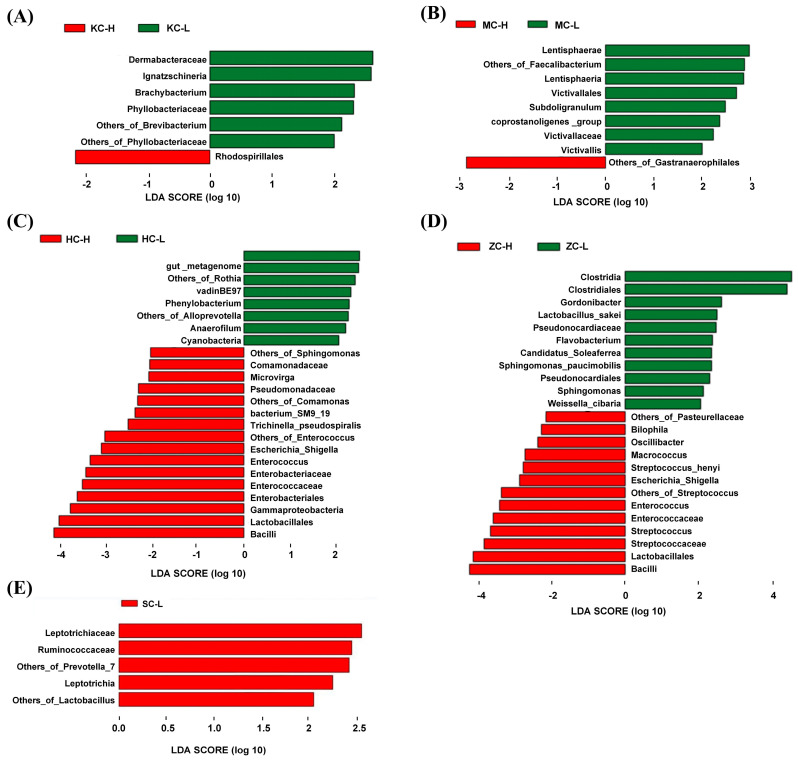
Intestinal flora differences between high and low abdominal fat rate groups: (**A**) Intestinal flora in the jejunum between high and low abdominal fat rate groups. (**B**) Intestinal flora in the cecum between high and low abdominal fat rate groups. (**C**) Intestinal flora in the ileum between high and low abdominal fat rate groups. (**D**) Intestinal flora in the rectum between high and low abdominal fat rate groups. (**E**) Intestinal flora in the duodenum between high and low abdominal fat rate groups.

Analysis of the differential flora between the intestinal segments using the LEfSe software identified the main flora specific to each intestinal segment. Significantly different species with an LDA score >2 were obtained (Figure 3). In the jejunum, Dermabacteraceae, *Ignatzschineria*, *Brachybacterium*, and Phyllobacteriaceae were abundant in the KC-L samples, while only Rhodospirillales was differentially enriched in KC-H samples. In the cecum, Lentisphaeria, Victivallales, *Subdoligranulum*, Victivallaceae, and eight other species were abundant in the MC-L, whereas Gastranaerophilales was differentially enriched in MC-H. In the ileum, VadinBE97, *Phenylobacterium*, *Anaerofilum*, and Cyanobacteria were abundant in HC-L, while Comamonadaceae, *Microvirga*, Pseudomonadaceae, *Enterococcus*, and Bacilli were abundant in HC-H. In the rectum, Clostridia, Clostridiales, *Gordonibacter*, *Flavobacterium*, Pseudonocardiales, and *Sphingomonas* were abundant in ZC-L, and Sphingomonas, *Bilophila*, *Oscillibacter*, *Macrococcus*, *Streptococcus*, and Bacilli were abundant in ZC-H. In the duodenum, only Leptotrichiaceae and *Leptotrichia* were abundant in the SC-L samples.

### 3.4. Community Function Prediction

**Figure 4:**
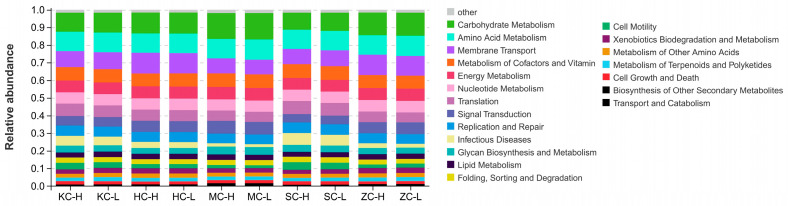
KEGG pathway functions of microorganisms in each intestinal segment.

The prediction of intestinal microbial function based on 16S rRNA gene sequencing results revealed that the top 20 pathways involved in the intestinal microbiota in F_2_ ducks were primarily related to metabolic processes (carbohydrate metabolism, tricarboxylic acid cycle, transport and catabolism, and amino acid and nucleotide metabolism), environmental information processing, membrane transport, cell motility, cell growth, and apoptosis (Figure 4). Microorganisms in the cecum were more enriched in the carbohydrate metabolism pathway than in the other intestinal segments, whereas SC sites were more enriched in infectious disease-related pathways. ZC and HC samples were considerably enriched in membrane transport pathways. Therefore, the cecum was predicted to play an important role in duck growth and development and may represent the main intestinal segment affecting abdominal fat deposition in ducks.

### 3.5. Cecal Microbial Diversity Analysis

**Figure 5:**
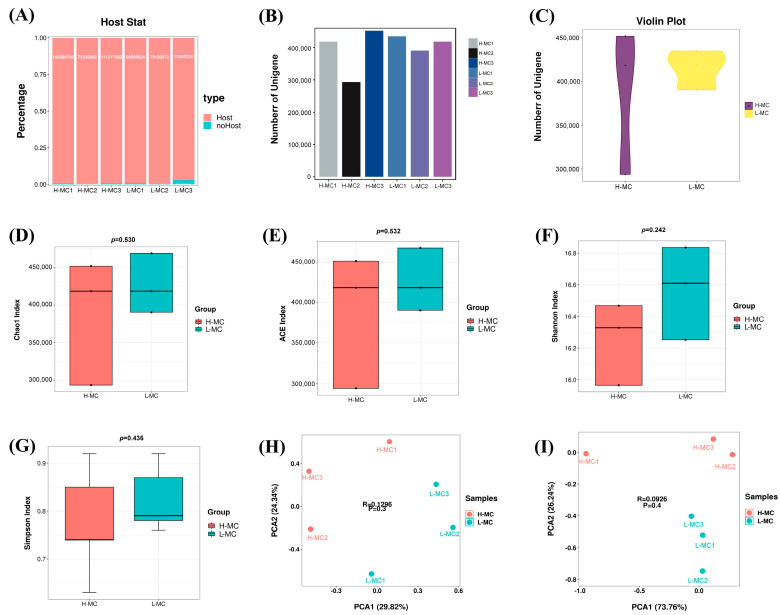
Metagenomic sequencing data analysis and cecal microbial diversity analysis: (**A**) Host and noHost ratio statistics after host sequence filtering. (**B**) Number of unigenes in each sample in the high and low abdominal fat rate groups. (**C**) Total number of unigenes in high and low abdominal fat rate groups. (**D**–**G**) Chao1, ACE, Shannon, and Simpson diversity indices in the high and low abdominal fat rate groups, respectively. (**H**) PCA index in high and low abdominal fat rate groups. (**I**) PCoA index in high and low abdominal fat rate groups.

Supplementary Table S4 presents the raw QC results obtained from metagenomic sequencing of the cecum from ducks in the HF and LF groups. Supplementary Figure S3 show the raw QC results of the macrogenome sequencing. The host genome was filtered to exclude its influence on the results (Figure 5A). Gene prediction results showed that L-MC samples contained more genes, while greater gene variation was detected in the H-MC samples (Figure 5B,C, Supplementary Table S5). The species diversity complexity was analyzed using the Chao1, ACE, Shannon, and Simpson indices (Figure 5D–G). Consistent with the 16S rRNA results, no significant differences were detected between the H-MC and L-MC groups in the microbial diversity of the cecal microflora (*p* > 0.05). In the β-diversity analysis of cecum microorganisms, PCA and PCoA were performed to visually determine the degree of difference in the bacterial flora structure between the two groups (Figure 5H,I); significant variability was detected between the two groups.

### 3.6. Cecal Microbial Indicator Species Analysis

**Figure 6:**
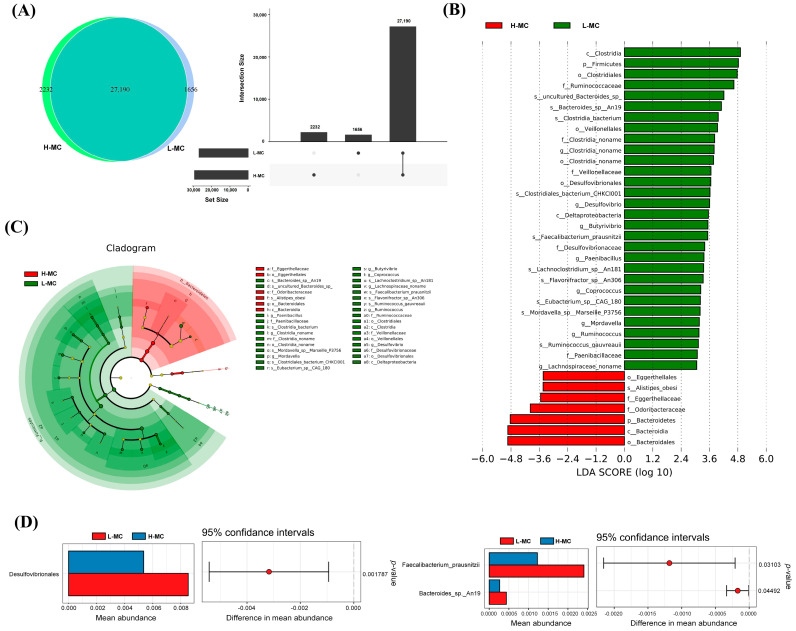
Cecal microbiota comparison between high and low abdominal fat rate groups: (**A**) Venn diagram of unique and shared cecal microbiota between high and low abdominal fat rate groups. (**B**) LEfSe analysis of cecal microbiota differences between high and low abdominal fat percentage groups. (**C**) Branch diagram of LEfSe analysis of cecal microbiota differences between high and low abdominal fat percentage groups. (**D**) Welch’s *t*-test analysis of cecal microorganisms that differ significantly between high and low abdominal fat rates.

Based on the Venn analysis, 2232 and 1656 endemic microorganisms were identified in the HF and LF groups, respectively, while 27,190 microorganisms were common to both groups (Figure 6A). Differences in predominant flora between groups were analyzed using LEfSe. *Paenibacillus*, *Butyrivibrio*, *Coprococcus*, Ruminococcaceae, Clostridiales, Veillonellaceae, and Firmicutes were core differentially abundant microflora. Meanwhile, Bacteroidetes and *Alistipes* were differentially enriched in the L-MC samples (Figure 6B,C). Subsequent analysis of differential flora between the two groups identified Desulfovibrionales, Faecalibacterium_prausnitzii, and Bacteroides_sp._An19 as being significantly more abundant in the L-MC group than in the L-MC group (Figure 6D).

### 3.7. Cecal Microbial Function Between the High and Low Abdominal Fat Rate Groups

**Figure 7:**
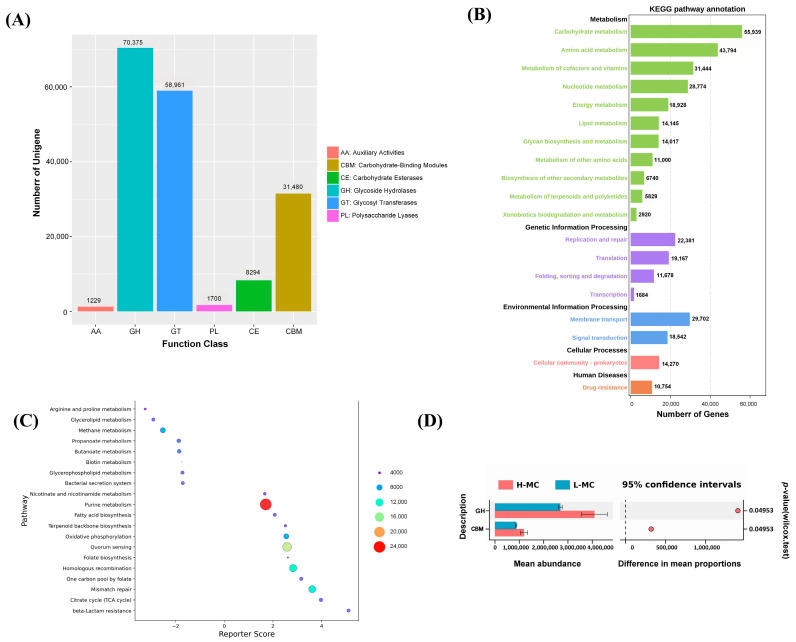
Functional analysis of the cecal microbiota in ducks with high and low abdominal fat rates: (**A**) Statistical graph after comparing and annotating Unigenes with the CAZy database. (**B**) Statistical graph after comparing and annotating Unigenes with the KEGG database. (**C**) Welch’s *t*-test analysis of the differential gene functions between high and low abdominal fat rates. (**D**) Differential analysis of metabolic pathways based on the reporter_score algorithm.

Genes were mapped to the CAZy database to obtain a functional abundance table, based on which statistical tests between the HF and LF groups were performed. The significantly enriched pathways included glycoside hydrolases (GHs), glycosyl transferases (GTs), polysaccharide lyases (PLs), carbohydrate activities (CEs), auxiliary activities (AAs), and carbohydrate-binding modules (CBMs) (Figure 7A). In addition, KEGG analysis was performed to obtain functional abundance for gene annotation; 431,255 genes were annotated in the KEGG pathway database (Figure 7B). These genes were primarily enriched in metabolic pathways such as carbohydrate, amino acid, nucleotide, and energy metabolism. They were also enriched in signaling pathways related to lipid and fat metabolism.

Functional differences in the differentially expressed genes were analyzed between the two groups (Figure 7D), revealing a higher abundance of genes related to GH and CBM in the H-MC samples than in the L-MC samples. Additionally, reporter score analysis of metabolic pathways (Figure 7C) identified significant differences in certain pathways related to metabolism, such as fatty acid biosynthesis and glycerophospholipid metabolism. This further confirmed that the intestinal microbiota affecting abdominal lipid deposition primarily impacted metabolism-related pathways.

### 3.8. Combined Metagenomic and Whole Transcriptome Analysis

**Figure 8:**
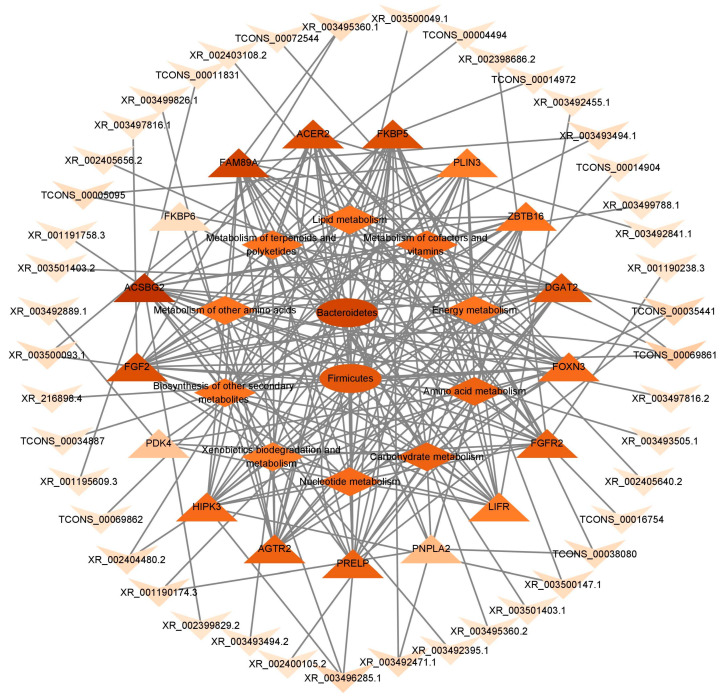
Metagenomic and whole transcriptome association analysis. Circles represent intestinal microorganisms; diamonds represent metabolic pathways; triangles represent genes; arrows represent lncRNAs. The darker the color, the stronger the correlation.

The cecal content macrogenome screened for differential flora among the groups based on key lncRNAs; genes were screened using a previously published whole transcriptome [6]. Only Firmicutes and Bacteroidetes were significantly associated with the whole-transcriptome results (Figure 8). Firmicutes and Bacteroidetes were associated with energy, lipid, and amino acid metabolism and with expression of *FGF2*, *FKBP5*, *PNPLA2*, *PLIN3*, *FGFR2*, *DGAT2*, and *ACER2*, which may affect abdominal lipid deposition. These genes were, in turn, affected by the regulation of XR_003493494.1, XR_003492471.1, XR_001190174.3, TCONS_00005095, XR_001190238.3, TCONS_00005095, XR_003492841.1, and other lncRNAs. Therefore, intestinal microbiota may influence the expression of lncRNA-targeted genes and, thus, abdominal lipid deposition.

## 4. Discussion

In 2002, intestinal microbiota were first shown to regulate host fat deposition as an environmental factor [12]. The composition of the intestinal microbiota may influence host fat storage [39,40] and impact the metabolic phenotype by producing high-energy substrates through fermentation, particularly short-chain fatty acids (SCFAs), acetate, butyrate, and propionate [41]. Recently, in a study on obese lean broiler lines, Jing et al. [42] found that intestinal microbiome–host interactions may contribute to fat deposition in chickens. Another study found that a relatively small group of different animal species shares intestinal microbiota [43]. However, the duck intestinal microbiota is regulated by multiple factors, including diet, environment, sex, and genetics, suggesting that different populations of the same species may have different intestinal microbiota [44,45].

Therefore, in this study, the intestinal microbiota of ducks with high and low abdominal fat content were explored to assess their effects on abdominal fat deposition in meat ducks. Consistent with Pechrkong et al. (2023) [46], who reported that Bacillus toyonensis supplementation in Barbary ducks increased the abundance of Ruminococcaceae and Bacteroidetes, associated with improved lipid metabolism and reduced intestinal inflammation, our findings also indicate that microbial taxa may play a role in modulating both fat deposition and host immune homeostasis. This underscores the broader physiological relevance of microbial composition beyond nutrient metabolism, suggesting that modulation of key microbial lineages can influence both energy balance and immunometabolic processes in ducks.

16S rRNA gene sequencing revealed that each intestinal segment (jejunum, ileum, cecum, rectum, and duodenum) contained abundant microbial communities. Alpha diversity analysis indicated that the regulatory effect on abdominal fat deposition was primarily attributed to bacterial community composition rather than species diversity. Among all segments, the cecum contained more microorganisms related to carbohydrate metabolism. These microorganisms likely contribute to SCFA production and energy extraction, thus affecting fat deposition. Consequently, the cecum was selected for metagenomic sequencing.

Indeed, previous studies indicate that increased abdominal fat accumulation is associated with reduced intestinal microbial diversity in poultry [47]. In the current study, we observed significant differences in cecal microbiota between HF and LF ducks. The LF group was enriched with beneficial taxa involved in carbohydrate fermentation and SCFA production, including Ruminococcaceae, Clostridiales, Veillonellaceae, and *Firmicutes*, whereas the HF group showed higher abundance of taxa potentially linked to dysbiosis, such as *Alistipes* and Eggerthellales. These results suggest that high abdominal fat content may reduce microbial diversity and alter functional microbial composition.

Beneficial microbes contribute to host lipid metabolism and energy homeostasis through SCFA production. SCFAs such as acetate, propionate, and butyrate act on G protein-coupled receptors on enteroendocrine cells, modulate lipogenic gene expression, and provide energy to enterocytes, linking microbial composition to host fat deposition [48,49]. Specific genera, including *Butyrivibrio* and *Coprococcus*, participate in carbohydrate fermentation and fatty acid metabolism, promoting butyrate synthesis and vitamin B production, which are important for host metabolic regulation [50,51,52]. Conversely, increases in taxa such as *Alistipes* may correlate with lipid accumulation and have been proposed as microbial markers of obesity [53,54]. Collectively, these findings indicate that cecal microbiota can directly or indirectly influence lipid synthesis and abdominal fat deposition, consistent with our multi-omics analysis.

Multi-omics association analysis demonstrated that two major microbial phyla, Firmicutes and Bacteroidetes, were significantly associated with host genes involved in adipogenesis and lipid metabolism, including FGF2, FKBP5, PNPLA2, PLIN3, FGFR2, DGAT2, ACER2, and several lncRNAs (XR_003493494.1, XR_003492471.1, XR_001190174.3, TCONS_00005095, XR_001190238.3, XR_003492841.1). These results suggest that cecal microbiota may influence host metabolic phenotype not only through energy extraction but also via transcriptional regulation of lipid metabolism pathways.

The Firmicutes/Bacteroidetes (F/B) ratio has been widely associated with lipid metabolism. In humans and mice, high-fat diets generally increase the abundance of Firmicutes and decrease Bacteroidetes, resulting in a higher F/B ratio [55]. In broilers, Xiang et al. [47] observed higher Firmicutes abundance in obese compared to lean chickens. However, in our study on ducks, both Firmicutes and Bacteroidetes were enriched in the HF and LF groups, with the F/B ratio showing species-specific patterns. This indicates that there are significant differences between different species. In ducks, the F/B ratio alone may not be a complete predictor of abdominal fat content. The interaction between various microbial groups and host genes may be a better indicator of fat deposition.

## 5. Conclusions

In this study, preliminary evidence of the contribution of the intestinal microbiota to abdominal fat deposition in ducks was provided by performing 16S rRNA gene sequencing and metagenome sequencing and evaluating previously published associations in the whole transcriptome. The results of 16S rRNA gene sequencing revealed that the cecum plays an essential role in duck growth and development and may be the main intestinal segment affecting abdominal fat deposition. The richness and diversity of microbial populations in the cecum decreased with the increasing accumulation of abdominal fat in ducks, with high abdominal fat decreasing the enrichment of beneficial bacteria and increasing that of pathogenic bacteria. In addition, Firmicutes and Bacteroidetes were significantly associated with differentially expressed lncRNAs and genes associated with high and low abdominal fat in the host, further suggesting that intestinal microbe–host interactions are associated with abdominal fat deposition in ducks.

## Figures and Tables

**Table 1:** Feed formulations and nutrient levels for ducks at different growth stages.

Ingredients (%)	0–22 d	23–42 d	Nutrient Composition	0–22 d	23–42 d
Corn	10.56	46.75	Metabolizable energy ^b^ (MJ/kg)	12.21	12.45
Wheat middlings	15.31	8.56	Crude protein ^a^ (g·kg^−1^)	18.8	17.3
Wheat bran	-	18.56	Crude fat ^b^ (g·kg^−1^)	2.8	3.6
Rice flour	35.02	-	Crude fiber ^b^ (g·kg^−1^)	6.0	7.0
Rice bran	15.52	3.23	Crude ash ^b^ (g·kg^−1^)	9.0	10.0
Peanut meal	-	3.12	Calcium ^b^ (g·kg^−1^)	1.1	1.1
Corn gluten meal	-	4.98	Phosphorus ^b^ (g·kg^−1^)	0.52	0.52
Soybean meal	12.62	6.01	Methionine ^b^ (g·kg^−1^)	0.42	0.31
Nucleotide-rich yeast	2.45	-	Lysine ^b^ (g·kg^−1^)	0.76	0.76
Limestone powder	1.46	1.78			
Dicalcium phosphate	1.06	1.01			
Compound premix	6	6			

Note: “a” denotes the nutrient content as a measured value; “b” denotes the nutrient content as a calculated value.

## Data Availability

The 16S rDNA and metagenomic raw sequence data have been submitted to NCBI SRA (SUB14345590 and SUB14347368), respectively.

## Supplementary Materials

The following supporting information can be downloaded at: https://www.mdpi.com/article/10.3390/ani15233393/s1, Supplementary Figure S1. Network plots showing the Pearson correlations among bacterial taxa. (A–F) Correlations at the phylum, class, order, family, genus, and species levels, respectively. The size of the dots corresponds to taxon abundance; a solid red line indicates a positive correlation, and a blue dotted line indicates a negative correlation. Supplementary Figure S2. Circos plots of species distribution. (A) Circos plots of species distribution at the phylum level; (B) Circos plots of species distribution at the class level; (C) Circos plots of species distribution at the order level; (D) Circos plots of species distribution at the family level; (E) Circos plots of species distribution at the genus level; (F) Circos plots of species distribution at the species level. Supplementary Figure S3. Base information statistical analysis after filtering. (A–C) Distribution of bases for the three samples with the high abdominal fat rate, respectively. (D–F) Distribution of bases for the three samples in low abdominal fat rate, respectively. Supplementary Table S1. All data processing statistics. Supplementary Table S2. Number of OTUs and Tags for different samples. Supplementary Table S3. Number of OTUs and Tags in different groups. Supplementary Table S4. Metagenomic Data Filtering Statistics. Supplementary Table S5. Gene prediction for each sample.

## References

[1] Ma J.S., Chang W.H., Liu G.H., Zhang S., Zheng A.J., Li Y., Xie Q., Liu Z.Y., Cai H.Y. (2015). Effects of flavones of sea buckthorn fruits on growth performance, carcass quality, fat deposition and lipometabolism for broilers. Poult. Sci..

[2] Demeure O., Duclos M.J., Bacciu N., Le Mignon G., Filangi O., Pitel F., Boland A., Boland S., Lagarrigue L.A., Cogburn J. (2013). Genome-wide interval mapping using SNPs identifies new QTL for growth, body composition and several physiological variables in an F2 intercross between fat and lean chicken lines. Genet. Sel. Evol..

[3] Ramiah S.K., Meng G.Y., Wei T.S., Keong Y.S., Ebrahimi M. (2014). Dietary Conjugated Linoleic Acid Supplementation Leads to Downregulation of PPAR Transcription in Broiler Chickens and Reduction of Adipocyte Cellularity. PPAR Res..

[4] Ge K., Ye P., Yang L., Kuang J., Chen X., Geng Z. (2020). Comparison of slaughter performance, meat traits, serum lipid parameters and fat tissue between Chaohu ducks with high-and low-intramuscular fat content. Anim. Biotechnol..

[5] Frank D., Watkins P., Ball A., Krishnamurthy R., Piyasiri U., Sewell J., Ortuño J., Stark J., Warner R. (2016). Impact of Brassica and Lucerne Finishing Feeds and Intramuscular Fat on Lamb Eating Quality and Flavor. A Cross-Cultural Study Using Chinese and Non-Chinese Australian Consumers. J. Agric. Food Chem..

[6] Yang C., Wang Z., Song Q., Dong B., Bi Y., Bai H., Jiang Y., Chang G., Chen G. (2022). Transcriptome Sequencing to Identify Important Genes and lncRNAs Regulating Abdominal Fat Deposition in Ducks. Animals.

[7] Munoz-Garach A., Diaz-Perdigones C., Tinahones F.J. (2016). Gut microbiota and type 2 diabetes mellitus. Endocrinol. Nutr..

[8] Turnbaugh P.J., Hamady M., Yatsunenko T., Cantarel B.L., Duncan A., Ley R.E., Sogin M.L., Jones W.J., Roe B.A., Affourtit J.P. (2009). A core gut microbiome in obese and lean twins. Nature.

[9] Org E., Lusis A.J. (2018). Using the natural variation of mouse populations to understand host-gut microbiome interactions. Drug Discov. Today Dis. Models.

[10] Ussar S., Fujisaka S., Kahn C.R. (2016). Interactions between host genetics and gut microbiome in diabetes and metabolic syndrome. Mol. Metab..

[11] Lee C.J., Sears C.L., Maruthur N. (2020). Gut microbiome and its role in obesity and insulin resistance. Ann. N. Y. Acad. Sci..

[12] Hooper L.V., Midtvedt T., Gordon J.I. (2002). How host-microbial interactions shape the nutrient environment of the mammalian intestine. Annu. Rev. Nutr..

[13] Backhed F., Ley R.E., Sonnenburg J.L., Peterson D.A., Gordon J.I. (2005). Host-bacterial mutualism in the human intestine. Science..

[14] Bäckhed F., Ding H., Wang T., Hooper L.V., Koh G.Y., Nagy A., Semenkovich C.F., Gordon J.I. (2004). The gut microbiota as an environmental factor that regulates fat storage. Proc. Natl. Acad. Sci. USA.

[15] Turnbaugh P.J., Ley R.E., Mahowald M.A., Magrini V., Mardis E.R., Gordon J.I. (2006). An obesity-associated gut microbiome with increased capacity for energy harvest. Nature.

[16] Ridaura V.K., Faith J.J., Rey F.E., Cheng J., Duncan A.E., Kau A.L., Griffin N.W., Lombard V., Henrissat B., Bain J.R. (2013). Gut microbiota from twins discordant for obesity modulate metabolism in mice. Science.

[17] Yang H., Xiang Y., Robinson K., Wang J., Zhang G., Zhao J., Xiao Y. (2018). Gut Microbiota Is a Major Contributor to Adiposity in Pigs. Front. Microbiol..

[18] Wen C., Yan W., Sun C., Ji C., Zhou Q., Zhang D., Zheng J., Yang N. (2019). The gut microbiota is largely independent of host genetics in regulating fat deposition in chickens. ISME J..

[19] Lyu W., Liu X., Lu L., Dai B., Wang W., Yang H., Xiao Y. (2021). Cecal Microbiota Modulates Fat Deposition in Muscovy Ducks. Front. Vet. Sci..

[20] Zhang X., Deng Y., Hu S., Hu X., Ma J., Hu J., Hu B., He H., Li L., Liu H. (2023). Comparative analysis of amino acid content and protein synthesis-related genes expression levels in breast muscle among different duck breeds/strains. Poult. Sci..

[21] Yang C., Li Y., Liu B., Chen A., Bai H., Jiang Y., Chang G., Chen G., Wang Z. (2025). Comparative analysis of duck meat quality in different breeds and age. Food Chem. X..

[22] Scanes C.G., Witt J., Ebeling M., Schaller S., Baier V., Bone A.J., Preuss T.G., Heckmann D. (2022). Quantitative Morphometric, Physiological, and Metabolic Characteristics of Chickens and Mallards for Physiologically Based Kinetic Model Development. Front Physiol..

[23] Wu Y., Ouyang J., Wang L., Hu J., Tang H., Zheng S., Xiong Y., Gao Y., Wu Y., Xiong R. (2025). Breed-specific gut microbiota and enterotype divergence in Chinese indigenous ducks. Front Microbiol..

[24] Zhang X., Akhtar M., Chen Y., Ma Z., Liang Y., Shi D., Cheng R., Cui L., Hu Y., Nafady A.A. (2022). Chicken jejunal microbiota improves growth performance by mitigating intestinal inflammation. Microbiom.

[25] Edgar R.C. (2013). UPARSE: Highly accurate OTU sequences from microbial amplicon reads. Nat. Methods..

[26] Ondov B.D., Bergman N.H., Phillippy A.M. (2011). Interactive metagenomic visualization in a Web browser. BMC Bioinform..

[27] Wang Q., Garrity G.M., Tiedje J.M., Cole J.R. (2007). Naive Bayesian classifier for rapid assignment of rRNA sequences into the new bacterial taxonomy. Appl. Environ. Microbiol..

[28] White J.R., Nagarajan N., Pop M. (2009). Statistical methods for detecting differentially abundant features in clinical metagenomic samples. PLoS Comput. Biol..

[29] Segata N., Izard J., Waldron L., Gevers D., Miropolsky L., Garrett W.S., Huttenhower C. (2011). Metagenomic biomarker discovery and explanation. Genome Biol..

[30] Aßhauer K.P., Wemheuer B., Daniel R., Meinicke P. (2015). Tax4Fun: Predicting functional profiles from metagenomic 16S rRNA data. Bioinformatics.

[31] Chen S.F., Zhou Y.Q., Chen Y.R., Gu J. (2018). fastp: An ultra-fast all-in-one FASTQ preprocessor. Bioinformatics.

[32] Langmead B., Salzberg S.L. (2012). Fast gapped-read alignment with Bowtie 2. Nat. Method..

[33] Li D., Liu C.M., Luo R., Sadakane K., Lam T.W. (2015). MEGAHIT: An ultra-fast single-node solution for large and complex metagenomics assembly via succinct de Bruijn graph. Bioinformatics.

[34] Zhu W.H., Lomsadze A., Borodovsky M. (2010). Ab initio gene identification in metagenomic sequences. Nucleic Acids Res..

[35] Fu L.M., Niu B.F., Zhu Z.W., Wu S.T., Li W.Z. (2012). CD-HIT: Accelerated for clustering the next-generation sequencing data. Bioinformatics.

[36] Buchfink B., Xie C., Huson D.H. (2015). Fast and sensitive protein alignment using DIAMOND. Nat. Methods.

[37] Huson D.H., Mitra S., Ruscheweyh H.J., Weber N., Schuster S.C. (2011). Integrative analysis of environmental sequences using MEGAN4. Genome Res..

[38] Huson D.H., Auch A.F., Qi J., Schuster S.C. (2007). MEGAN analysis of metagenomic data. Genome Res..

[39] Park S., Ji Y., Jung H.Y., Park H., Kang J., Choi S.H., Shin H., Hyun C.K., Kim K.T., Holzapfel W.H. (2017). Lactobacillus plantarum HAC01 regulates gut microbiota and adipose tissue accumulation in a diet-induced obesity murine model. Appl. Microbiol. Biotechnol..

[40] Suárez-Zamorano N., Fabbiano S., Chevalier C., Stojanović O., Colin D.J., Stevanović A., Veyrat-Durebex C., Tarallo V., Rigo D., Germain S. (2015). Microbiota depletion promotes browning of white adipose tissue and reduces obesity. Nat. Med..

[41] Flint H.J., Bayer E.A., Rincon M.T., Lamed R., White B.A. (2008). Polysaccharide utilization by gut bacteria: Potential for new insights from genomic analysis. Nat. Rev. Microbiol..

[42] Jing Y., Yuan Y., Monson M., Wang P., Mu F., Zhang Q., Na W., Zhang K., Wang Y., Leng L. (2022). Multi-Omics Association Reveals the Effects of Intestinal Microbiome-Host Interactions on Fat Deposition in Broilers. Front. Microbiol..

[43] Huang P., Zhang Y., Xiao K., Jiang F., Wang H., Tang D., Liu D., Liu B., Liu Y., He X. (2018). The chicken gut metagenome and the modulatory effects of plant-derived benzylisoquinoline alkaloids. Microbiome.

[44] Diaz Carrasco J.M., Casanova N.A., Fernández Miyakawa M.E. (2019). Microbiota, Gut Health and Chicken Productivity: What Is the Connection?. Microorganisms.

[45] Gao R., Zhu C., Li H., Yin M., Pan C., Huang L., Kong C., Wang X., Zhang Y., Qu S. (2018). Dysbiosis Signatures of Gut Microbiota Along the Sequence from Healthy, Young Patients to Those with Overweight and Obesity. Obesity.

[46] Pechrkong T., Incharoen T., Hwanhlem N., Kaewkong W., Subsoontorn P., Tartrakoon W., Numthuam S., Jiménez G., Charoensook R. (2023). Effect of *Bacillus toyonensis* BCT-7112T supplementation on growth performance, intestinal morphology, immune-related gene expression, and gut microbiome in Barbary ducks. Poult. Sci..

[47] Xiang H., Gan J.K., Zeng D.S., Li J., Yu H., Zhao H.Q., Yang Y., Tan S.W., Li G., Luo C.W. (2021). Specific Microbial Taxa and Functional Capacity Contribute to Chicken Abdominal Fat Deposition. Front. Microbiol..

[48] Bouter K.E., van Raalte D.H., Groen A.K., Nieuwdorp M. (2017). Role of the Gut Microbiome in the Pathogenesis of Obesity and Obesity-Related Metabolic Dysfunction. Gastroenterology.

[49] Fan Y., Pedersen O. (2021). Gut microbiota in human metabolic health and disease. Nat. Rev. Microbiol..

[50] Enjalbert F., Combes S., Zened A., Meynadier A. (2017). Rumen microbiota and dietary fat: A mutual shaping. J. Appl. Microbiol..

[51] Nogal A., Louca P., Zhang X., Wells P.M., Steves C.J., Spector T.D., Falchi M., Valdes A.M., Menni C. (2021). Circulating Levels of the Short-Chain Fatty Acid Acetate Mediate the Effect of the Gut Microbiome on Visceral Fat. Front. Microbiol..

[52] Wong J.M., de Souza R., Kendall C.W., Emam A., Jenkins D.J. (2006). Colonic health: Fermentation and short chain fatty acids. J. Clin. Gastroenterol..

[53] Yin J., Li Y., Han H., Chen S., Gao J., Liu G., Wu X., Deng J., Yu Q., Huang X. (2018). Melatonin reprogramming of gut microbiota improves lipid dysmetabolism in high-fat diet-fed mice. J. Pineal. Res..

[54] Louis S., Tappu R.M., Damms-Machado A., Huson D.H., Bischoff S.C. (2016). Characterization of the Gut Microbial Community of Obese Patients Following a Weight-Loss Intervention Using Whole Metagenome Shotgun Sequencing. PLoS ONE.

[55] Zheng X.J., Huang F.J., Zhao A.H., Lei S., Zhang Y.J., Xie G.X., Chen T.L., Qu C., Rajani C., Dong B. (2017). Bile acid is a significant host factor shaping the gut microbiome of diet-induced obese mice. BMC Biol..

