# Supplementary figures and images for: Multi-Omics Insights into the Relationship Between Intestinal Microbiota and Abdominal Fat Deposition in Meat Ducks
Source: Animals (Basel). 2025 Nov 24;15(23):3393. doi: 10.3390/ani15233393 (PMC12691082; doi:10.3390/ani15233393)

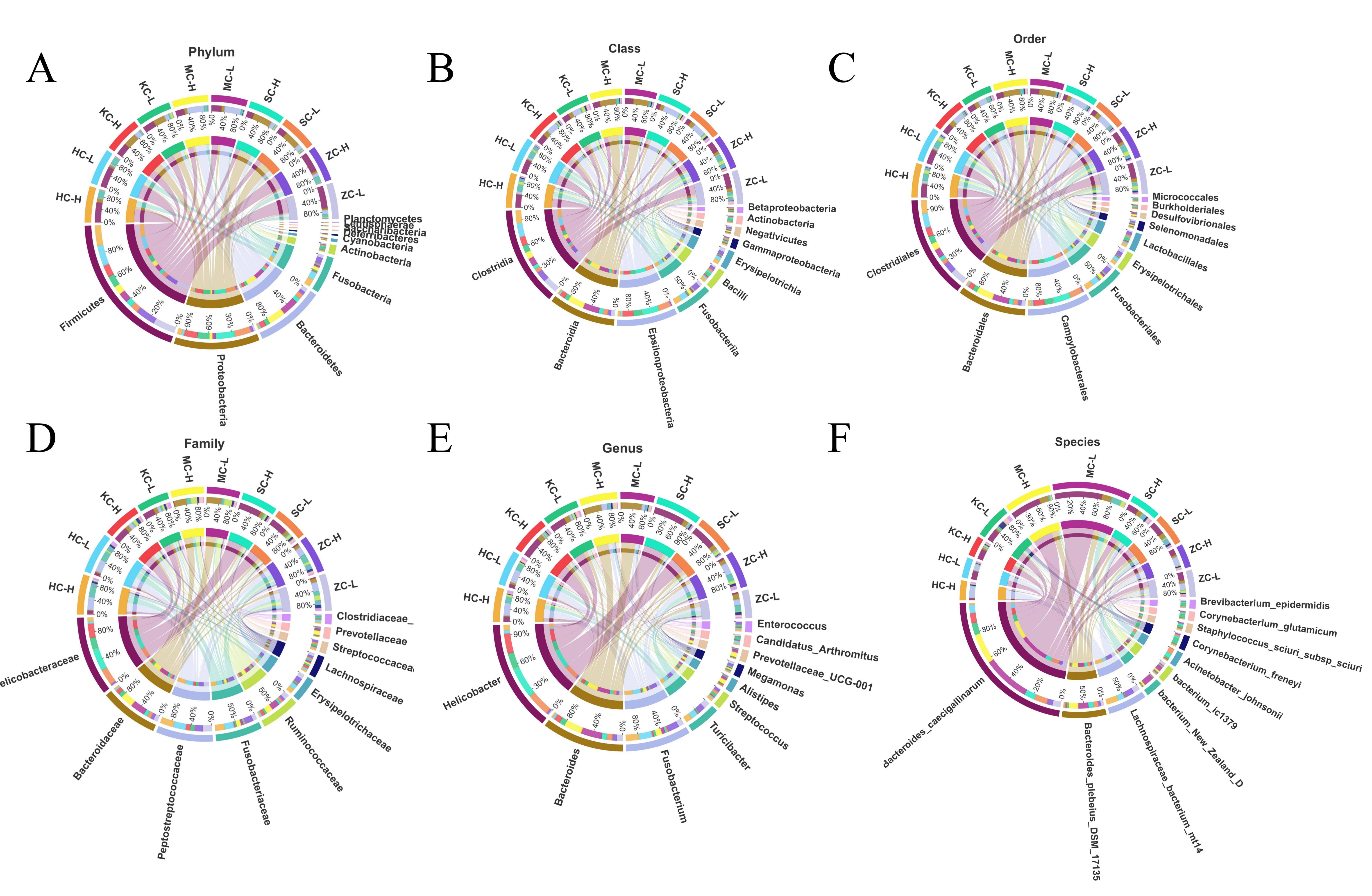

Supplement: Supplementary file 1 [file animals-15-03393-s001.zip › Supplementary Figure S1.jpg]

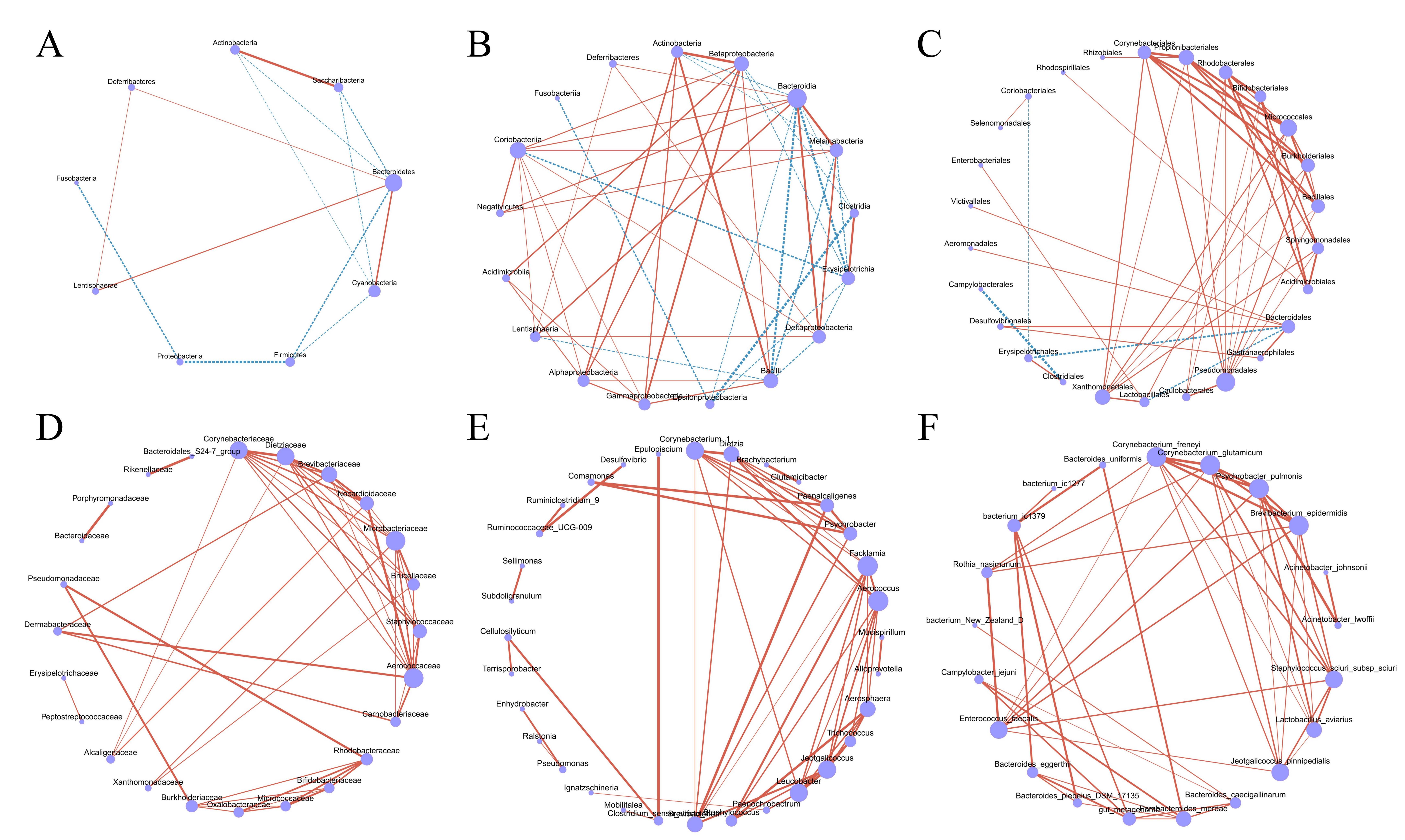

Supplement: Supplementary file 1 [file animals-15-03393-s001.zip › Supplementary Figure S2.jpg]

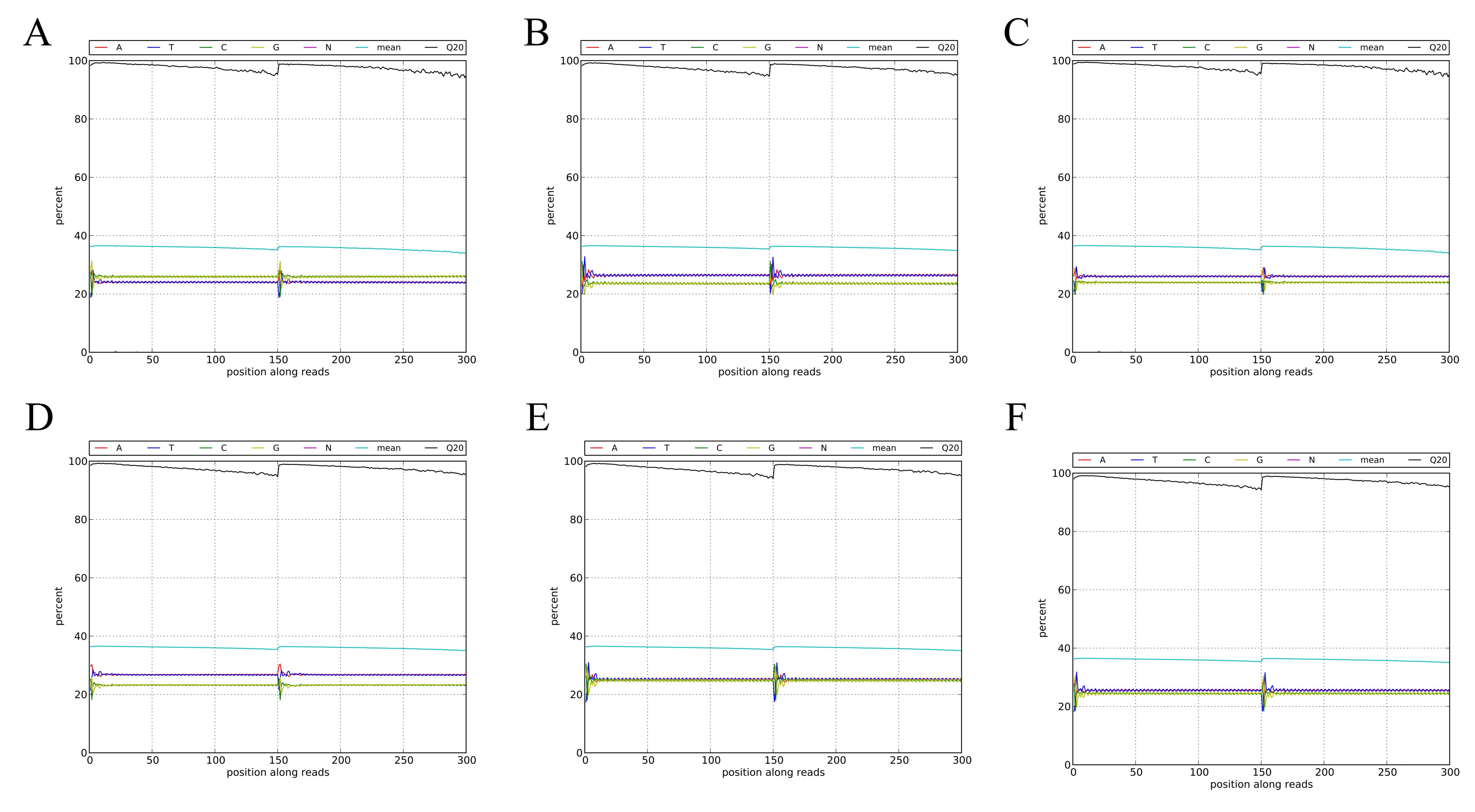

Supplement: Supplementary file 1 [file animals-15-03393-s001.zip › Supplementary Figure S3.jpg]
